# Editorial for the Special Issue “Parasitic Diseases from Wild Animals with Emphasis on Zoonotic Infections”

**DOI:** 10.3390/microorganisms9112267

**Published:** 2021-10-31

**Authors:** María Teresa Gómez-Muñoz

**Affiliations:** Department of Animal Health, Faculty of Veterinary Sciences, University Complutense of Madrid, 28040 Madrid, Spain; mariateg@ucm.es

**Keywords:** zoonotic parasites, wildlife, *Toxoplasma*, *Cryptosporidium*, *Giardia*, *Blastocystis*, *Leishmania*, *Echinococcus*, *Anisakis*

## Abstract

The present Editorial intends to briefly describe the findings published in the Special Issue, “Parasitic diseases from wild animals with an emphasis on zoonotic infections”. Prevalence data or diagnostic techniques were the focus of several zoonotic parasites transmitted from wildlife, including the protozoa *Toxoplasma*, *Cryptosporidium*, *Giardia*, *Blastocystis* and *Leishmania*, and the helminths *Echinococcus* and *Anisakis.*

Human activity greatly affects wildlife by displacement due to urbanization or exotic travelling, for example, but the availability of food and shelter is also a reclaim to wild animals to peri-urban areas. In this context of human activity, there is a magnified contact between humans and wildlife. In addition, global warming enlarges the number of vectors and water intake and subsequently increases disease transmission through those routes.

There are many pathogens, including viruses, bacteria and fungus, and many parasites affecting wild animals that may spill over to humans if adequate conditions are present. While domestic animals are under veterinary supervision, and transmission is less probable, wild animals are not, and human infections derived from contact with wild fauna or from certain epidemiological situations occur.

Zoonotic parasites from wild animals are less frequently studied than viruses or bacteria. Some of them are vector-borne transmitted (e.g., *Leishmania*), while others infect their host by ingestion of contaminated food, including meat (e.g., *Toxoplasma*), fish (e.g., *Anisakis*), vegetables (e.g., *Echinococcus*) or drinking water (e.g., *Giardia*, *Cryptosporidium*). Among zoonotic parasites affecting wild animals, protozoa are the most frequent and commonly studied, such as *Toxoplasma*, *Cryptosporidium*, *Giardia*, *Blastocystis* or *Leishmania*, included in this Special Issue.

*Toxoplasma* is a ubiquitously distributed parasite that infects many species of animals and supposes a threat to immune-compromised people. The transmission of *Toxoplasma* through meat consumption, among other routes, is a frequent one, and game species, such as wild boar and deer, are frequent reservoirs for humans after being hunted [1]. The detection of *Toxoplasma* is afforded using several techniques: while direct detection (acid pepsin digestion, magnetic enrichment, and RT-PCR) reflects a present infection, indirect methods offer information on past infections, and bioassays can confirm the infectivity of DNA positive samples. In this context, rabbits and hares are scarcely studied, but several studies suggest the importance of these animals in *Toxoplasma* infection [2,3]. Seroprevalence in rabbits (MAT, IHA, IFAT, ELISA) varies from 0.9 to 37.5%, while in hares (MAT, IHA, IFAT), it ranges from 0–21%. PCR has been less frequently employed, and prevalence values of domestic rabbits and hares are lower (2.1–16.2%) than seroprevalence values, but a high percentage of infection was found in clinical cases (100%) or in hunted wild animals from Portugal (67.9%) [3]. This is relevant since wild rabbits are a source of food in many countries. On four occasions, viable and infective cysts from *Toxoplasma* have been obtained from rabbits belonging to three different genotypes, although not much is known so far on the genetic diversity of *Toxoplasma* detected and characterized from rabbits and hare. Raptors are examined as reservoirs of *Toxoplasma*, and they can act as indicators of environmental contamination by the parasite. A study carried out in Italy found 62.5% of brain samples positive to at least one out of three parasite markers [4] and added the common kestrel (*Falco tinnunculus*) as a new host for the parasite.

Wild rodents are less explored than other animal species as carriers of fecal zoonotic parasites, such as *Cryptosporidium* and *Giardia* spp., probably due to the lower amount of feces they excrete. However, they can live in high densities in critical locations, such as water supplies or production fields [5]. In fact, *Cryptosporidium* isolated from deer mice and yellow-bellied marmot from California displayed DNA sequences that were 99.75–100% identical to zoonotic species, such as *C. parvum*, *C. ubiquitum* and *C. xiaoi* [5].

Wild rodents are also infected with zoonotic assemblages of *Giardia duodenalis* (A and B). Since most of them carry non-zoonotic species, such as *G. microti* and *G. muris*, this may hinder the detection of *G. duodenalis*. However, a new workflow protocol has been developed to improve the situation and, when applied, was able to detect and genotype 36.4% of the samples that could not be previously characterized [6].

*Blastocystis* is a gastrointestinal protist with a controversial role in gastrointestinal disease in humans. The genetic diversity of *Blastocystis* is large, with 25 subtypes described (ST1-ST19, ST21, ST23-ST29). There are not many studies on the role of wild animals as reservoirs for *Blastocystis* to humans (ST1-ST9 and ST12) and domestic animals, but one of them reported a high percentage of white-tailed deer infected (88.8%), most of them with mixed subtype infections, and twelve subtypes were identified. The studied white-tailed deer carried zoonotic subtypes ST1, ST3 or ST4 (8.5%) and other subtypes that can affect domestic animals (ST10, ST14, ST21, ST23-ST26) [7]. Moreover, two novel subtypes were described and validated from white-tailed deer (ST30 and ST31).

Among the protozoa, *Leishmania* is widely explored since leishmaniosis is one of the neglected diseases with almost a global distribution. Several species of *Leishmania* are zoonotic, and most of them were reported in wild animals. Ten animal orders, including 189 species, were infected by the genus [8]. *L. infantum*, the most worldwide distributed, was described in 98 species, while *Leishmania* (*Viannia*) was found in 52 different species. *L. mexicana*, *L. amazonensis*, *L. major* and *L. tropica* were more restricted geographically. The orders Carnivora and Rodentia are the most relevant hosts for *L. infantum* and the genus *Leishmania* (*Viannia*), with some animals showing lesions but most of them asymptomatic. Among Carnivora, invasive species, such as the American mink (*Neovison vison*), were found infected with *L. infantum* in Spain, an endemic country in several regions, at large percentages (90.1%) [9]. There are different PCR protocols to detect the presence of the parasite, but the most sensitive targets for PCR were the repeat region, the kinetoplast region and the large subunit of the DNA [9].

Besides protozoans, several zoonotic helminths may infect wild animals. In this case, the presence of *Echinococcus ortleppi* (genotype G5) was described as infecting a wild boar for the first time in Portugal. This finding has public health implications since these animals are hunted and consumed by humans and accessible to the hunting dogs [10].

Finally, there is a need for a holistic approach to understanding diseases provoked by pathogens, including environmental factors, co-infections and co-morbidities, and inter-animal and human–animal interactions that produce changes in the prevalence and severity of the diseases. In this context, an unusual paper relating the microbiota that a zoonotic parasite, such as *Anisakis*, can inoculate to humans is included in this Special Issue [11].

## Data Availability

Not applicable.
